# Treatment Resistant First-Episode Psychosis in an Autistic Adolescent Male

**DOI:** 10.1192/bjo.2026.11853

**Published:** 2026-06-30

**Authors:** Abdulgafar Adebayo Yusuf, Harshini Prasanna Kumar, Deniz Yazici, Aram Yaseri

**Affiliations:** Birmingham Women’s and Children’s NHS Foundation trust, Birmingham, United Kingdom

## Abstract

**Aims:**

Drug resistant first episode psychosis in a 17-year-old adolescent male with autism spectrum disorder presents with a significant diagnostic and therapeutic challenge especially with associated physical comorbidities. Medication intolerance, persistently low white cell count, partial response and early relapse after discharge remain the prominent risks. This case highlights the complex nature of therapeutic decision making and discharge planning in a high-risk young person.

**Methods:**

A 17-year-old adolescent male who was admitted to the in-patient CAMHS unit with a 14-month history of progressive psychotic symptoms characterised by chronic auditory and visual hallucinations, paranoid delusions; alleging his parents to be impostors; depressed mood, anhedonia, and social withdrawal. With a diagnosis of autism spectrum disorder, history of significant trauma, years of school bullying and street robbery. He has a past medical history of cardiac arrest with unknown aetiology and on treatment with implantable cardioverter defibrillator.

During initial admission, he was polite, compliant with treatment although with partial insight. Family support was robust, and leave was utilized with no significant adverse incidents. Despite participation in ward activities his affect remained predominantly flat with persisting psychotic symptoms albeit with less intensity. Pharmacological management remained challenging, initially commenced on aripiprazole, titrated to 15mg which was discontinued due to significant restless legs symptoms requiring procyclidine. Olanzapine was initiated and gradually increased to a maximum of 12.5 mg with partial stabilization necessitating discharge to the community.

Weeks following discharge, worsening complex delusions and commanding hallucinations and high-risk behaviours in the community was noted. These include fire-setting and possession of weapons.

This led to re-admission and risperidone was trialled and gradually titrated to 5 mg with marked elevation of prolactin with minimal improvement.

Giving treatment resistance, clozapine was considered and is being approached cautiously due to his history of cardiac arrest, persistently low white cell counts and hyperprolactinemia with close psychiatric and cardiology consultation followed with gradual risperidone taper.

**Results:**

This case illustrates the complexity in managing treatment resistant first-episode psychosis in autistic adolescent with significant physical comorbidities.

**Conclusion:**

Careful antipsychotic selection, robust discharge planning, and intensive multidisciplinary follow-up are essential to reduce relapse and serious harm.

